# Asynchrony of Apical Polarization, Luminogenesis, and Functional Differentiation in the Developing Thyroid Gland

**DOI:** 10.3389/fendo.2021.760541

**Published:** 2021-12-17

**Authors:** Ellen Johansson, Shawn Liang, Carmen Moccia, Therese Carlsson, Daniel Andersson, Henrik Fagman, Mikael Nilsson

**Affiliations:** ^1^ Sahlgrenska Center for Cancer Research, Institute of Biomedicine, University of Gothenburg, Göteborg, Sweden; ^2^ Department of Laboratory Medicine, Institute of Biomedicine, University of Gothenburg, Göteborg, Sweden; ^3^ Department of Clinical Pathology, Sahlgrenska University Hospital, Göteborg, Sweden

**Keywords:** thyroid development, embryonic growth, cell polarity, folliculogenesis, mucin 1, pericentrin, Nkx2-1, thyroglobulin

## Abstract

Follicular thyroid tissue originates from progenitors derived from a midline endodermal primordium. Current understanding infers that folliculogenesis in the embryonic thyroid designates the latest morphogenetic event taking place after the final anatomical shape and position of the gland is established. However, this concept does not consider the fact that the thyroid isthmus develops chronologically before the lobes and also contains all progenitors required for lobulation. To elucidate whether cells committed to a thyroid fate might be triggered to differentiate asynchronously related to maturation and developmental stage, mouse embryonic thyroid tissues from E12.5-17.5 were subjected to immunofluorescent labeling of biomarkers (progenitors: NKX2-1; differentiation: thyroglobulin/TG); folliculogenesis: E-cadherin/CDH1; luminogenesis: mucin 1/MUC1; apical polarity: pericentrin/PCNT; basement membrane: laminin; growth: Ki67), quantitative RT-PCR analysis (*Nkx2.1*, *Tg*, *Muc1*) and transmission electron microscopy. *Tg* expression was detectable as early as E12.5 and gradually increased >1000-fold until E17.5. *Muc1* and *Nkx2.1* transcript levels increased in the same time interval. Prior to lobulation (E12.5-13.5), MUC1 and TG distinguished pre-follicular from progenitor cells in the developing isthmus characterized by intense cell proliferation. Luminogenesis comprised redistribution of MUC1+ vesicles or vacuoles, transiently associated with PCNT, to the apical cytoplasm and the subsequent formation of MUC1+ nascent lumens. Apical polarization of pre-follicular cells and lumen initiation involved submembraneous vesicular traffic, reorganization of adherens junctions and ciliogenesis. MUC1 did not co-localize with TG until a lumen with a MUC1+ apical membrane was established. MUC1 delineated the lumen of all newly formed follicles encountered in the developing lobes at E15.5-17.5. Folliculogenesis started before establishment of a complete follicular basal lamina. These observations indicate that embryonic thyroid differentiation is an asynchronous process consistent with the idea that progenitors attaining a stationary position in the connecting isthmus portion undergo apical polarization and generate follicles already at a primordial stage of thyroid development, i.e. foregoing growth of the lobes. Although the thyroid isthmus eventually comprises minute amounts of the total thyroid volume and contributes little to the overall hormone production, it is of principal interest that local cues related to the residence status of cells – independently of a prevailing high multiplication rate – govern the thyroid differentiation program.

## Introduction

In organ development, it appears important that fate-committed progenitors maintain an undifferentiated state until the required morphogenetic stages and final destination of cells are accomplished before terminal differentiation takes place. Immature embryonic cells are mainly occupied with two basic features: replication to generate a sufficient pool of cells, and migration to colonize and shape the many different parts of the embryo. Precocious differentiation may thus conflict with cells abilities to proliferate and migrate, and to attain their correct numbers and final positions in developing tissues. Mechanisms promoting the embryonic progenitor state *in vivo* are about to be deciphered, for example, in the developing lungs Sox9 prevents inappropriate differentiation during growth accompanying branching morphogenesis (1, 2). Inactivation of Sox9 thus not only prematurely differentiates bronchial cells but also confers lung hypoplasia, which suggests that embryonic growth and differentiation at the cellular level are mutually exclusive processes.

Suppressed differentiation of a progenitor cells pool likely commences postnatally until obtaining the final organ size. Nonetheless, postnatal growth merely relies on diminishing amounts of proliferating cells, outnumbered by the expansion of differentiated cells. It is likely although not formally proven that adult stem cells derive from and retain at least some features of the embryonic progenitors that belongs to the same lineage. Difficulties to characterize and delineate the pool of organ-specific progenitor cells in intact embryos are inherited to the fact that for most parenchymal organs the progenitors are multipotent and give rise to several distinct cell types that are specified and generated under the influence of distinct although yet poorly characterized spatiotemporal cues. This infers that the allocation of a specific cell lineage is further divided by the commitment of subpopulations of progenitors to specific fates, e.g. towards ducts and acini in developing glandular tissues. Thus, as illustrated by new findings from single cell analysis of mouse pancreatic development (3, 4), at a given developmental stage genuine progenitors occur side by side with terminally differentiated cells admixed with cells that possess transitional phenotypes.

In thyroid development, progenitors arising in anterior endoderm sequentially migrate and proliferate before differentiating into a single endocrine cell type, the follicular epithelial cells. This is accomplished by distinct morphogenetic steps in which the thyroid primordium buds off from the pharyngeal floor and bifurcates to form the final anatomical shape of the gland, two lobes connected across the midline by an isthmus, identical in mice and humans (5). Terminal differentiation comprising *de novo* expression of thyroid-specific genes and the generation of follicles, a unique multicellular modality for efficient biosynthesis and storage of thyroid hormones, is considered to be late events in thyroid development, accompanied by cessation of proliferation of the cells that are about to differentiate (6). Recently, we found that the mouse thyroid, foregoing the differentiation stage, adopts a branching program of morphogenesis presumably reflecting the evolutionary origin of an exocrine thyroid ancestor (7). The embryonic thyroid thus provides a simplified model for investigating spatiotemporal regulation of fate decisions in a uniform progenitor cell type. In this study, we show that some/a few thyroid progenitors – contrary to current views and concepts – start to functionally differentiate and form microfollicles in the prospective isthmus already before lobulation takes place. Notably, luminogenesis at this time and location coincides with the highest proliferation rate of all thyroid developmental stages. Moreover, lumen initiation involving apical relocalization of Mucin 1 (MUC1) does not require apical accumulation and secretion of thyroglobulin (TG), suggesting that folliculogenesis and expression of thyroid-specific genes involved in hormone production – although being functionally linked – are independently regulated processes *in vivo*.

## Materials and Methods

### Animals and Tissue Sampling

Wildtype embryos of C57BL/6 mice were collected at embryonic days E12.5, E13.5, E14.5, E15.5 and E17.5, respectively. The morning when a vaginal plug could be observed was considered as E0.5. Each developmental stage required surgical considerations for tissue sampling; in general, embryonic thyroid tissue was excised *en bloc* with adjacent neck structures and further microdissected under microscope to minimize damage by handling. Each analysis was reproduced on specimens from different litters, comprising 3–6 embryos per age including both sexes. Animal experiments were approved by the regional ethic committee (Dnr 26-2013 and Dnr 5.8.18-03925/2018) according to European standards and national regulations provided by the Swedish Agriculture Agency.

### Immunofluorescence

Embryos were immersion-fixed with 4% paraformaldehyde overnight at 4°C and washed three times in PBS (pH 7.3) before cryoprotection in 30% sucrose overnight at 4°C, embedding in Tissue Tek compound (Sakura, Zoeterwoude, Netherlands) and freezing at -80°C for storage. Serial horizontal cryosections (thickness 10 µm) were collected on Superfrost plus glass slides (Mentzel Gläser, Germany), permeabilized with 0.1% Triton X-100 in PBS for 20 min, and blocked with 2% normal donkey serum (Jackson) in PBS for one hour before incubation with primary antibodies, diluted in blocking buffer, overnight at 4°C. Primary antibodies were: rabbit anti-NKX2-1 (1:1000; Cat. No. PA0100, Biopat Srl., Sant´ Angelo a Cupolo, Italy); rat anti-E-cadherin/CDH1 (ECCD-2; 1:500; Cat. No. 13-1900, ThermoFischer Scientific, Waltham, MA); rabbit anti-TG (1:1000; DAKO, Glostrup, Denmark); rabbit-anti-laminin/LAM (1:500; Cat. No. L9393, Sigma-Aldrich, St Louis, MO); rabbit anti-pericentrin/PCNT (1:1000; Cat. No. ab4448, Abcam, Cambridge, UK; Armenian hamster anti-mucin1/MUC1 (1:200 of CT2 monoclonal against aa 239-255 (SSLSYTNPAVAATSANL) from the cytoplasmic tail of MUC1; gift from Cathy Madsen at Sandra Gendler Lab, Mayo Clinic. The CT2 antibody is also available at: ThermoFischer, Cat. No. MA5-11202). Secondary antibodies in blocking buffer were added for 60 min followed by incubation with streptavidin-FITC for 30 min, both at room temperature. Secondary antibodies were: Rhodamine Red-X-conjugated donkey anti-rabbit IgG (Jackson Immunoresearch Laboratories Inc. West Grove, PA, USA); Rhodamine Red-X-conjugated donkey anti-goat IgG (Jackson); biotin-conjugated donkey anti-rat IgG (Jackson) and biotin-conjugated donkey anti-goat IgG (Jackson) followed by Streptavidin-FITC (DAKO). CT2 staining used a FITC-conjugated goat anti-Armenian hamster-IgG (1:200; Cat. No. 127-095-160, Jackson). Sections were counterstained with DAPI (Sigma-Aldrich, Taufkirchen, Germany) and finally mounted with Fluorescence Mounting Medium (DAKO). Each incubation step was ended with washing 3 x 5 min with 0.1% Triton X-100 in PBS. Images were captured using a Zeiss Axioscope 2 Plus fluorescence microscope equipped with a Nikon DS-Qi1Mc camera and processed with NIS Element Imaging.

### Electron Microscopy

E13.5-14.5 embryos were immersion-fixed with 2% formaldehyde and 2.5% glutaraldehyde dissolved in 0.05 M sodium cacodylate buffer containing 0.02% sodium azide for 24 h. An oscillation tissue slicer was used to produce, from the embryo’s neck, 150 µm transverse sections that were postfixed in 1% osmium tetroxide with 1% potassium hexacyanoferrate for 2 h, at 4°C, and thereafter contrasted *en bloc* with 0.5% uranyl acetate for 1 h in the dark at room temperature. After stepwise dehydration in alcohol series and acetone, specimens were embedded in Agar 100 Resin according to standard procedures. Overview sections (thickness: 1 µm) were cut on a Leica EM UC6 ultramicrotome and stained with azure methylene blue for light microscopic identification of thyroid primordia that were further processed. Ultrathin sections (thickness: approximately 60 nm) were contrasted with uranyl acetate and lead citrate, coated with carbon by evaporation, and examined and photographed with a LEO 912AB Omega transmission electron microscope.

### Quantitative Real-Time PCR

Embryonic thyroid tissue derived from different developmental stages was stored at -80°C in RNA later (Thermo Fisher Scientific). Samples obtained at E12.5-13.5 comprised a standardized embryonic neck slice including the thyroid primordium and surrounding tissues, whereas E14.5-E17.5 samples consisted of microdissected thyroid lobes. Thawed samples were homogenized with Tissuelyser II (Qiagen) and the RNA was extracted using the RNeasy plus micro kit (Qiagen) according to manufacturer’s instructions. Final RNA amounts were determined spectrophotometrically (NanoDrop1000; Thermo Scientific). Complementary DNA (cDNA) was generated in duplicate using TATAA GrandScript cDNA Synthesis Kit (TATAA Biocenter) in 10 µl reactions containing 40 to 200 ng total RNA on a Veriti 96-well Thermal Cycler (Applied Biosystems by Thermo Fisher Scientific) according to manufacturer’s instructions and stored at -20°C. All cDNA samples were diluted with 90 µl nuclease-free water. Quantitative real-time PCR was performed using a CFX384 Touch Real-Time PCR Detection System (Bio-Rad Laboratories) in 6 µl reactions containing 1x TATAA SYBR GrandMaster Mix (TATAA Biocenter), 400 nM of each primer and 2 µl diluted cDNA. The temperature profile used was 95°C for 2 min, followed by 46 cycles of amplification: 95°C for 5 s, 60°C for 30 s, 70°C for 20 s, and melting curve analysis: 65°C to 95°C, increment 0.5°C for 5 s. All samples were verified by melting curve analysis. Cycles of quantification (Cq) values were determined by regression using Bio-Rad CFX Maestro 2.0 (version 5.0.021.0616; Bio-Rad Laboratories). Missing data were replaced by cDNA replicate or maximum observed Cq value +1, whereafter Cq values larger than 35 were replaced by 36 and Cq values normalized to total RNA levels. Relative quantification using reference genes was applied. Five potential reference genes from Reference Gene Panel Mouse (*Actb*, *B2m*, *Gapdh*, *Pgk1* and *Ppia*; TATAA Biocenter) and in addition *Pax8* and *Nkx2.1* were evaluated with NormFinder (8). *Gapdh* and *Pax8* were chosen as optimal reference genes for time-dependent (E12.5-17.5) comparison. Following normalization to reference genes, Cq values were transformed to relative quantities and log2 transformed. Finally, the expression levels of double negative samples were manually set to half of the lowest observed value (*i.e.*: -1 on log2 scale). Data preprocessing was performed using GenEx (version 7.1.12.224; MultiD Analyses) and all experiments were conducted in accordance with the Minimum Information for Publication of Quantitative Real-Time PCR Experiments (MIQE) guidelines (9). Primers (Sigma-Aldrich) for *Tg* (NCBI reference sequence NM_009375.2; forward: 5’-GTG AGC TAC AAA GAG AGA AGG C-3’; reverse: 5’-TGG CAG AAG GAC AGA CAA ACT-3’), *Pax8* (NCBI reference sequence NM_011040.4; forward: 5’-TCA GGG CGA GAG ATG GTG G-3’; reverse: 5’-AGG AGG AAT ACG GGG TGT GG-3’), *Nkx2.1* (NCBI reference sequence NM_009385.3; forward: 5’-CAG CCT ATC CCA TCT GAA CTC C-3’; reverse: 5’-CGG GCG AAT GGT GGT CTT TG-3’) and *Muc1* (NCBI reference sequence NM_013605.2; forward: 5’- TAC CAC ACT CAC GGA CGC TA-3’; reverse: 5’-TGG GGT GAC TTG CTC CTA CA-3’) were designed and had specificity validated as previously described (10).

### Statistics

Statistical analysis was performed using IBM SPSS Statistics software (version 27; IBM). Expression levels of *Tg*, *Muc1* and *Nkx2.1* were compared using the non-parametric Kruskal-Wallis test with obtained p-values adjusted by the Bonferroni correction for multiple tests. p-values below 0.05 were considered significant.

## Results

### Mucin 1 Delineates Lumen Formation Designating *De Novo* Folliculogenesis in the Embryonic Thyroid Gland

**Figure 1:**
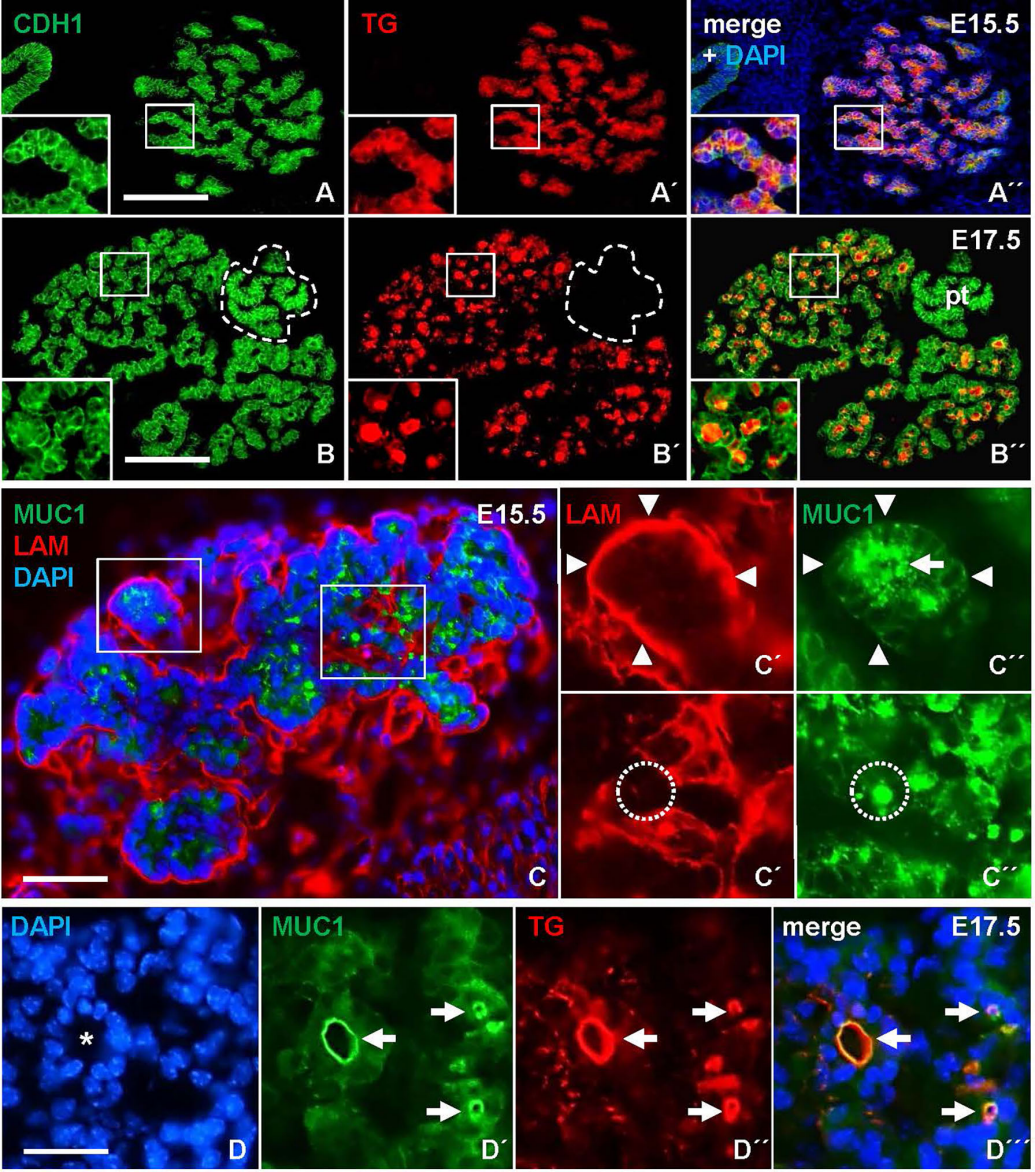
Mucin 1 (MUC1) redistribution during *de novo* follicle formation in the embryonic thyroid gland in mice. Transverse sections of one of the thyroid lobes. **(A-A´´)** Ubiquitous expression of thyroglobulin (TG) confined to the cytoplasm at E15.5. Inset shows detail of parenchymal branch. **(B-B´´)** TG concentrated in follicle lumens at E17.5. Inset shows detail of follicular parenchyma. **(C-C´´)** MUC1 expression at E15.5. Insets **(C´-C´´)** show MUC1 concentrated in apical cytoplasm (arrow) and microlumen (encircled). Arrowheads indicate basal lamina of parenchymal branch. **(D-D´´´)** Co-localization of TG and MUC1 (arrows) at luminal border of a large follicle (asterisk) at E17.5. Note smaller follicles show similar co-staining. pt, parathyroid (encircled); CDH1, E-cadherin; LAM, laminin; DAPI, nuclear stain. Bars: 150 µm **(A, B)**, 50 µm **(C)** and 25 µm **(D)**.

Terminal differentiation of mouse thyroid progenitors is considered to take place after organogenesis essentially is completed and the bilobed gland already gained its orthotopic position. We first investigated this process by monitoring the expression and distribution of TG, the thyroid prohormone, in the prospective lobes. At E15.5, the majority of parenchymal cells showed strong TG immunoreactivity that was largely confined to the cytoplasm (**Figures 1A-A´´**). Two days later, TG was mainly present in the lumens of small follicles that were prevalent in all parts of the parenchyma (**Figures 1B-B´´**). This suggested that follicles were generated more or less synchronously – accompanying the functional differentiation of cells – throughout the tissue in late mouse thyroid development.

Next, we examined whether MUC1, a luminal glycoprotein that is expressed at the apical membrane in mucosal and glandular organs (11) including the thyroid (12), might be instrumental in capturing key features of lumen biogenesis during *de novo* follicle formation in the embryonic thyroid. Sections were additionally co-stained for laminin (LAM) to determine the orientation of progenitor cells presumed to undergo apicobasal polarization during folliculogenesis. This showed that, at E15.5, MUC1 was expressed already at a pre-follicular stage, and displayed a mostly granular cytoplasmic staining located centrally in the parenchymal cords that were outlined peripherally – i.e. on the outer border – by a continuous LAM+ basement membrane (**Figures 1C, C´-C´´**, upper panel). Notably, MUC1 also labeled structures with resemblance of a conspicuous lumen presumably formed by the assembly of MUC1+ vesicles or vacuoles (**Figures 1C, C´-C´´**, lower panel). As evident at E17.5, luminogenesis involving MUC1 was confirmed by colocalization with TG at the apical surface of nascent follicles (**Figures 1D-D´´´**). This also revealed coinciding MUC1+ lumens of various size and presumed maturity. MUC1 immunostaining thus enabled us to distinguish apical polarization and lumen formation representing distinct – and probably sequential – phases of folliculogenesis.

### Mucin1 and Thyroglobulin Co-Expression Reveals Early Differentiation of Cells in the Primordial Thyroid Isthmus

**Figure 2:**
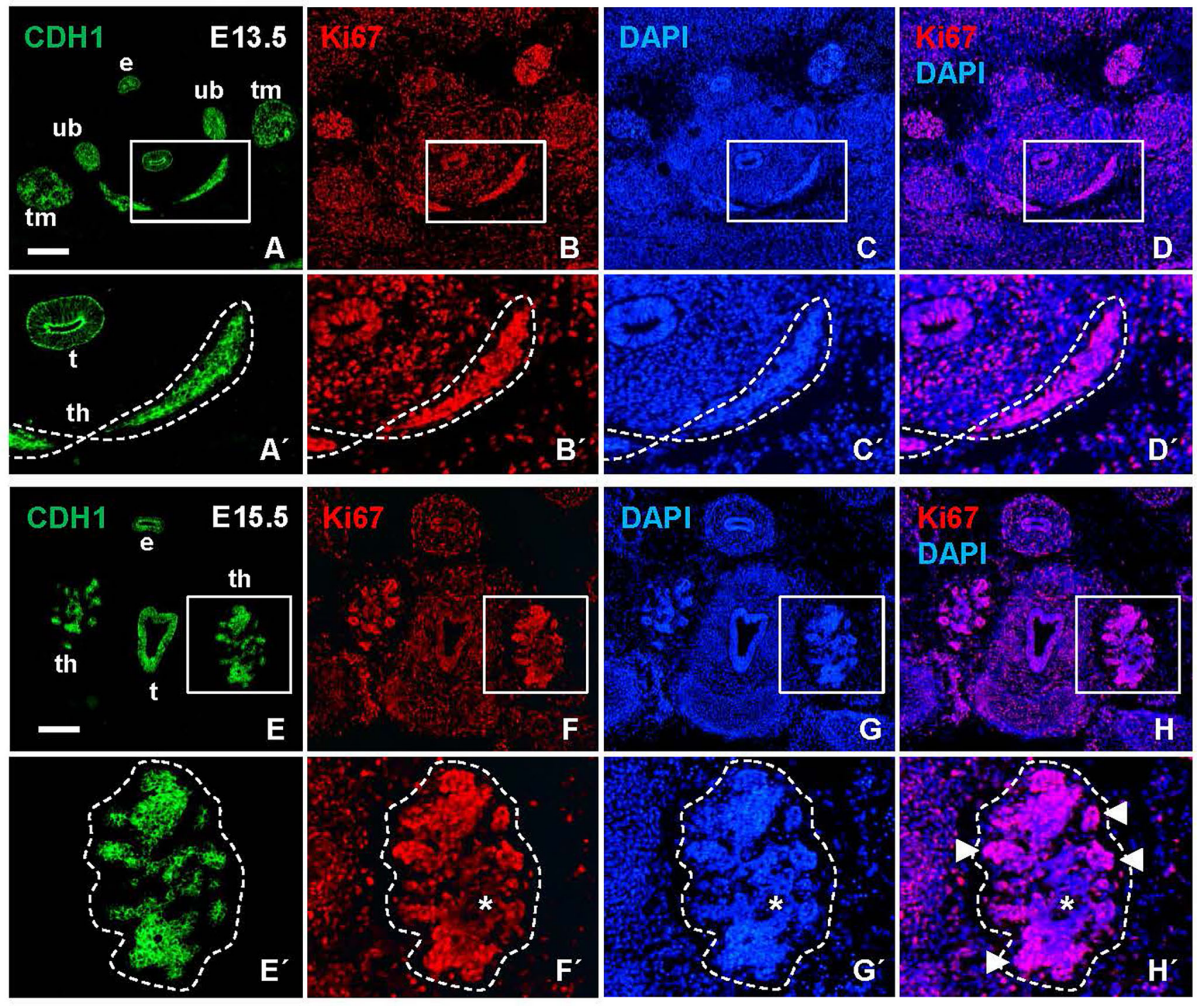
Proliferation status of the developing mouse thyroid prior to and during lobulation. Representative images of Ki67 labeling in transverse sections obtained from E13.5 and E15.5 embryos. E-cadherin (CDH1) was co-labeled for identification of epithelial tissues. **(A–D)** Prospective thyroid isthmus at E13.5. **(A´-D´)** show high power of boxed areas in A-D and with the thyroid primordium encircled. **(E–H)** Thyroid lobes emerging bilaterally at E15.5. **(E´-H´)** show high power of boxed areas in **(E–H)** and with the left lobe encircled. Arrowheads indicate Ki67+ branch tips; asterisks indicate Ki67 negative lobe interior. th, thyroid; t, trachea; e, esophagus; ub, ultimobranchial body (bilaterally); tm, thymus (bilateral primordia); DAPI, nuclear stain. Bars: 300 µm.

**Figure 3:**
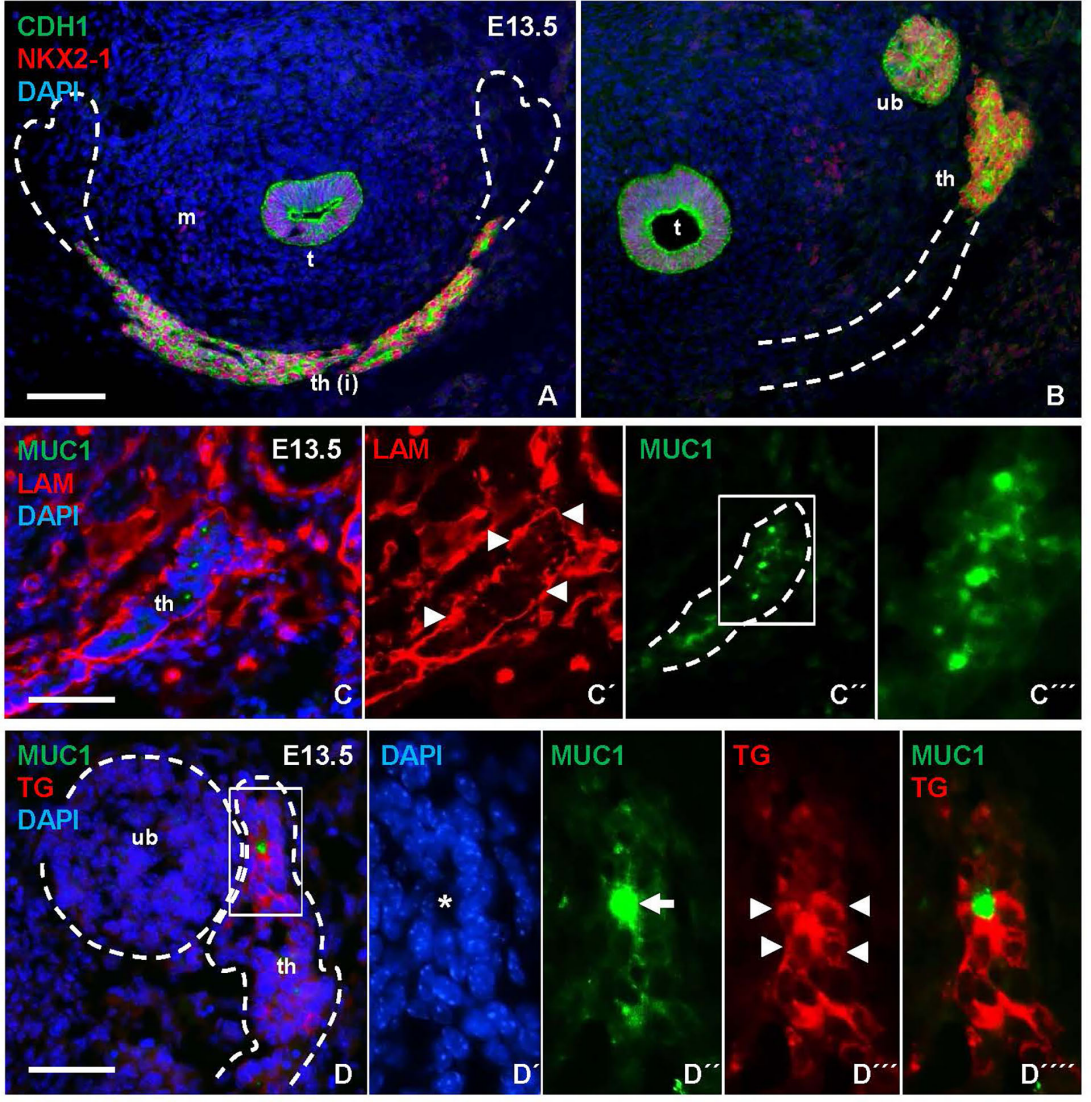
Mucin 1 (MUC1) expression associated with premature differentiation of NKX2-1+ thyroid progenitors. Data obtained at E13.5 prior to thyroid lobe formation. **(A, B)** Midline thyroid primordium in front of trachea. Broken lines indicate thyroid portions present in A and B, respectively, in serial sections of the same specimen. **(C-C´´´)** MUC1 expression in prospective thyroid isthmus. **(C)** is merge image of C´ and C´´. Arrowheads in **(C´)** indicate basal lamina. Broken line (in **C´´**) indicates outer border of primordium. **(C´´´)** shows high magnification of inset in **(C´´)**. **(D-D´´´´)** Thyroglobulin (TG) expression in thyroid primordium. Broken lines in **(D)** indicate outer borders of thyroid and ultimobranchial body approaching. **(D-D´´´´)** show a newly formed follicle (asterisk) with a central MUC1+ microlumen (arrow) and TG in the apical cytoplasm of follicular cells (arrowheads) corresponding to inset in **(D)** t, trachea; th, thyroid; th(i), thyroid isthmus (prospective); ub, ultimobranchial body; m, mesenchyme; CDH1, E-cadherin; LAM, laminin. Bars: 100 µm **(A)** and 50 µm **(B, C)**.

The heterogeneous MUC1 distribution among cells and follicles within the same tissue portions argued that this feature of thyroid differentiation was less synchronous than suggested by the more even TG staining pattern in late thyroid development. To further address the possibility of asynchronous differentiation, earlier developmental stages were investigated. Prior to lobe formation, it is assumed that cells maintain an undifferentiated state as the midline thyroid primordium is subjected to rapid growth and enlargement (7). However, this notion is merely conceptual rather than supported by experimental findings other than at E13.5 the majority of primordial cells are cycling (**Figures 2A–D, A´-D´**) whereas at E15.5 the lobe parenchyma consists of growth-arrested cells centrally and proliferating cells peripherally (**Figures 2E–H, E´-H´**), reflecting a typical proximal-distal feature of branching growth (7). Notably, most thyroid progenitors present at E13.5 will be retained to reside in the prospective isthmus portion that passes across the midline in front of the trachea (**Figure 3A**) and eventually connects the left and right lobes of the gland; it is only the lateral-most cells of the primordium that establish contact with the paired ultimobranchial bodies (**Figure 2B**) and that will give rise to all follicular cells of the lobes (7). The obvious question is whether or not the isthmus cells resist precocious differentiation and mature at a later stage i.e. simultaneously with the lobe cells.

**Figure 4:**
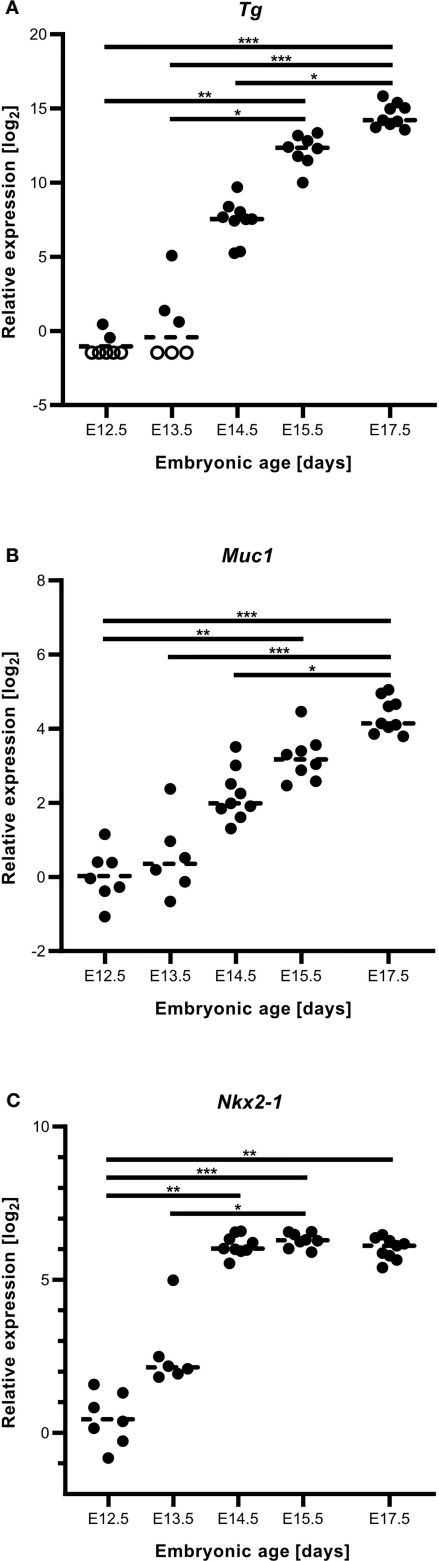
Gene expression accompanying thyroid differentiation and luminogenesis in the developing mouse thyroid. Reverse transcription quantitative real-time PCR analysis of tissue samples obtained at E12.5-E17.5. **(A)** Thyroglobulin (*Tg*), **(B)** mucin 1 (*Muc1*) and **(C)** *Nkx2.1* transcript levels. Each dot corresponds to an individual sample and empty circles to expression levels below limit of detection. Dashed lines correspond to mean expression. *p ≤ 0.05; **p ≤ 0.01; ***p ≤ 0.001. Comparison was optimized using *Gapdh* and *Pax8* in combination as reference genes in all instances.

As shown in **Figures 3B, C**, numerous MUC1+ cells occurred in the midline thyroid primordium outlined by a continuous LAM+ sheet. Notably, MUC1 was not evenly distributed but accumulated at multiple sites with a separating distance of several cell diameters consistent with formation of nascent lumens (**Figures 3C–C´´´**). TG immunostaining confirmed that many of these MUC1+ thyroid cells indeed were triggered to functionally differentiate (**Figures 3D–D´´´´**). To corroborate this finding, embryonic thyroid tissue samples were microdissected from mouse embryos between E12.5 and E17.5, and the extracted RNA was processed for quantitative real-time PRC (qPCR) analysis. This showed that *Tg* transcripts were detectable in some samples and *Muc1* expression was evident in all samples already at E12.5-E13.5 (**Figures 4A, B**). Both genes showed progressively elevated levels in the following days (E14.5-E17.5): *Tg* increased >1000-fold whereas the enhanced expression of Muc1 was more modest. Intermediate expression levels were monitored between E13.5-E14.5 coinciding with fusion of the thyroid primordium with the ultimobranchial bodies. Altogether, this indicated that some embryonic thyroid cells differentiated much earlier than expected i.e. before the start of lobulation.

We also monitored the expression of *Nkx2.1* and *Pax8* in the same samples. *Pax8* showed the most stable mRNA levels and in fact served as the most suitable reference gene jointly with *Gapdh*. Notably, *Nkx2.1* gradually increased as the primordium transited to the lobulation stage after which transcript levels were more stable during late thyroid development (**Figure 4C**). Immunostaining has previously revealed that NKX2-1 and PAX8 are ubiquitously expressed in thyroid progenitor cells in all developmental stages (7), and that both genes are required for proper thyroid development (13). The present results from qPCR analysis suggests that altered relative expression levels of *Nkx2.1* and *Pax8* might play a role in the differentiation process.

### Luminogenesis and Thyroglobulin Secretion Are Sequential and Independently Regulated Processes

**Figure 5:**
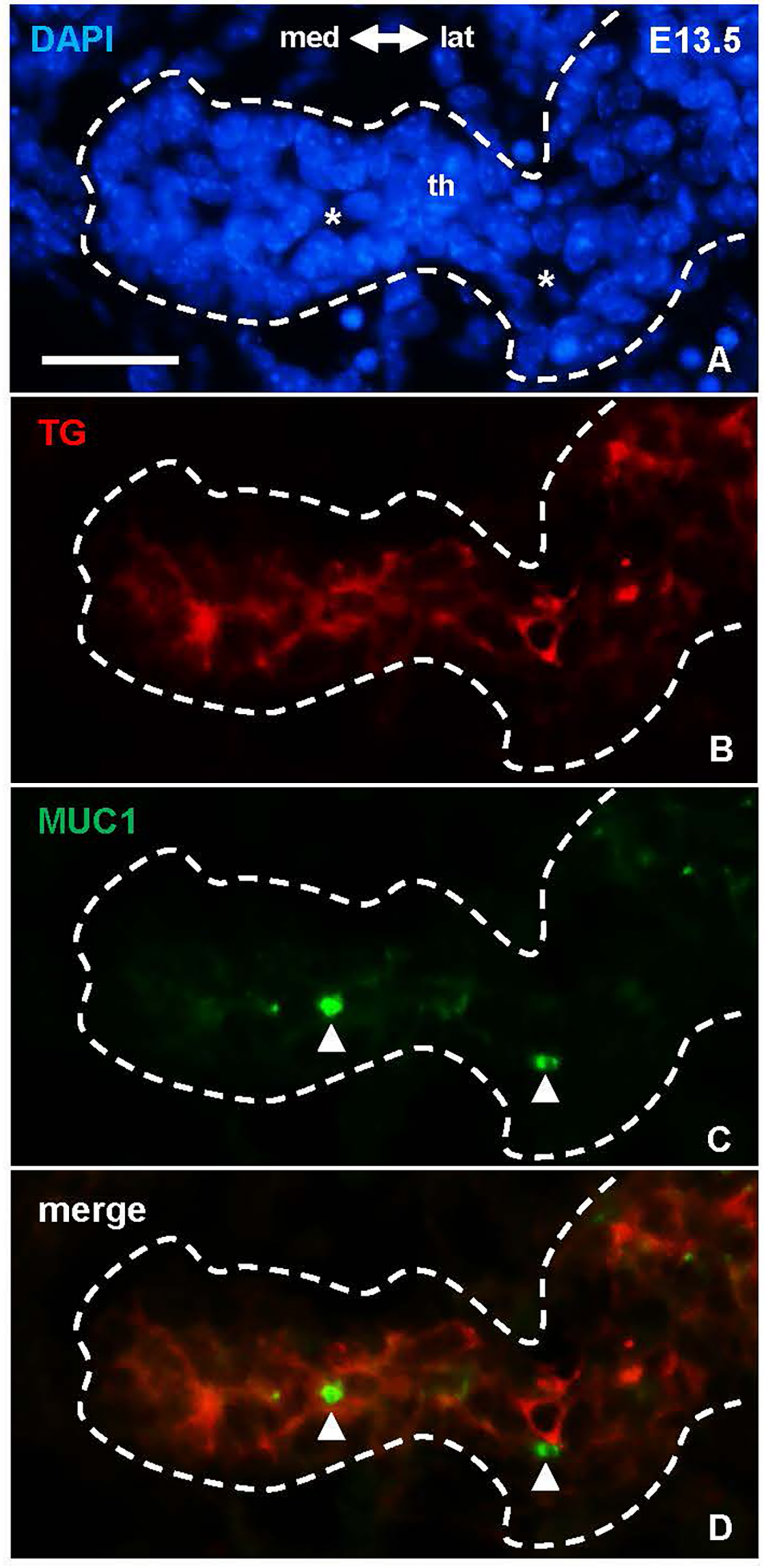
Dissociated distribution of mucin 1(MUC1) and thyroglobulin (TG) in the prospective thyroid isthmus. **(A–D)** Midpiece of the thyroid primordium (th) at E13.5. Asterisks in **(A)** indicate nucleus-free areas corresponding the MUC1+ structures (arrowheads) in **(B)** Note MUC1 does not co-localize with cytoplasmic TG. med, medial direction; lat, lateral direction. Bar: 25 µm.

There is ample evidence from cell culture studies indicating that the molecular machinery required for thyroid hormone synthesis can occur in unpolarized cells that do not form follicles and, *vice versa*, that thyroid-derived epithelial cells lacking any signs of functional differentiation retain the ability to generate follicles (14). Whether these processes are similarly independent *in vivo* has not previously been studied. As expected, in the developing lobes MUC1 and TG mostly co-localized in newly formed follicles (**Figures 1D´-D´´´**), which at this stage predominated in the tissue and made it difficult to discern any possible pre-follicular dissociation of the two markers. However, immunolocalization at E13.5 revealed that MUC1 and TG mostly had a different subcellular distribution in the prospective isthmus (**Figures 5A–D**). Specifically, TG immunoreactivity were more widely distributed whereas MUC1 concentrated in rounded structures located in widened areas between cells that likely corresponded to sites of luminogenesis (**Figures 5A, C**). Notably, such MUC1+ structures were devoid of TG (**Figure 5D**), consistent with a delay in transfer of TG to the lumen about to be formed. This also showed that TG expression was heterogeneous as TG+ cells resided mostly interiorly in the primordium whereas peripheral cells were essentially devoid of TG (**Figures 5A, B**). The prospective isthmus thus consisted of both progenitor cells retaining an immature state and cells undergoing functional differentiation, and these two cell populations were at least partly segregated. These observations further suggested that luminogenesis and TG secretion into the nascent follicle lumen are differentially regulated processes.

**Figure 6:**
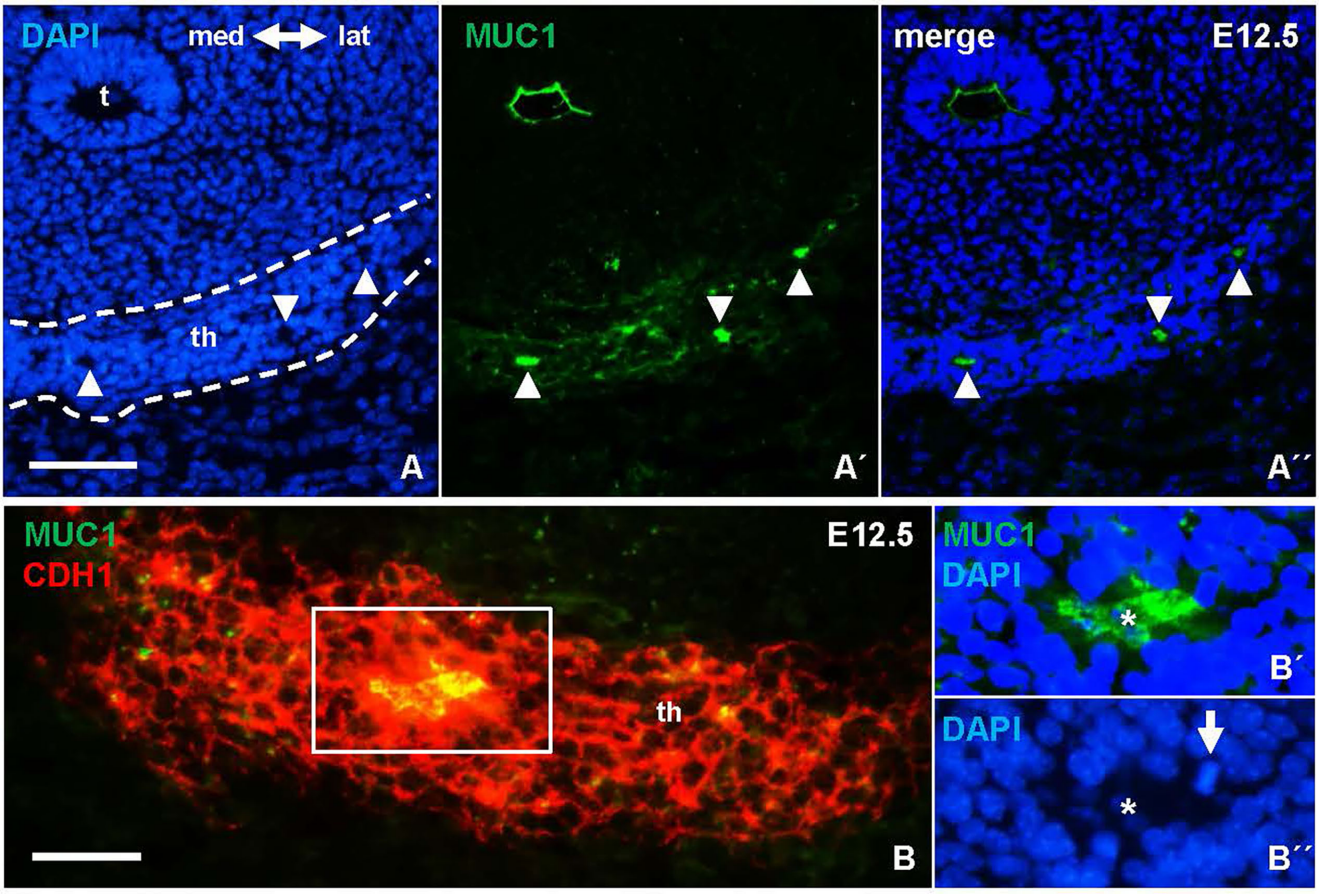
Earliest signs of folliculogenesis in the thyroid primordium delineated by mucin 1 (MUC1) expression. Representative images from E12.5; the prospective thyroid isthmus is outlined. **(A-A´´)** MUC1+ lumen formations (arrowheads). **(B-B´´´)** Epithelial cells surrounding a MUC1+ lumen. **(B´, B´´)** show boxed area with separated MUC1 and DAPI stainings. Arrow indicates mitotic cell (metaphase stage) facing the lumen (asterisk). th, thyroid; t, trachea; med, medial direction; lat, lateral direction; CDH1, E-cadherin. Bars: 100 µm **(A)** and 50 µm **(B)**.

Notably, MUC1 distribution suggestive of luminogenesis was evident also at E12.5 (**Figures 6A-A´´**). Follicles present at this developmental stage occasionally exhibited mitotic profiles consistent with oriented cell division perpendicular to the apicobasal axis of the lining epithelium (**Figures 6B-B´´**), which thus likely contributed to follicle enlargement. Folliculogenesis thus takes place once the descent of the midline primordium is finished and concomitantly with progenitor cells starting to proliferate intensely to form the thyroid isthmus (6, 7).

### Ultrastructural Evidence of Apical Vesicle Trafficking Towards Cell-Cell Junctions Foregoing Follicle Formation

**Figure 7:**
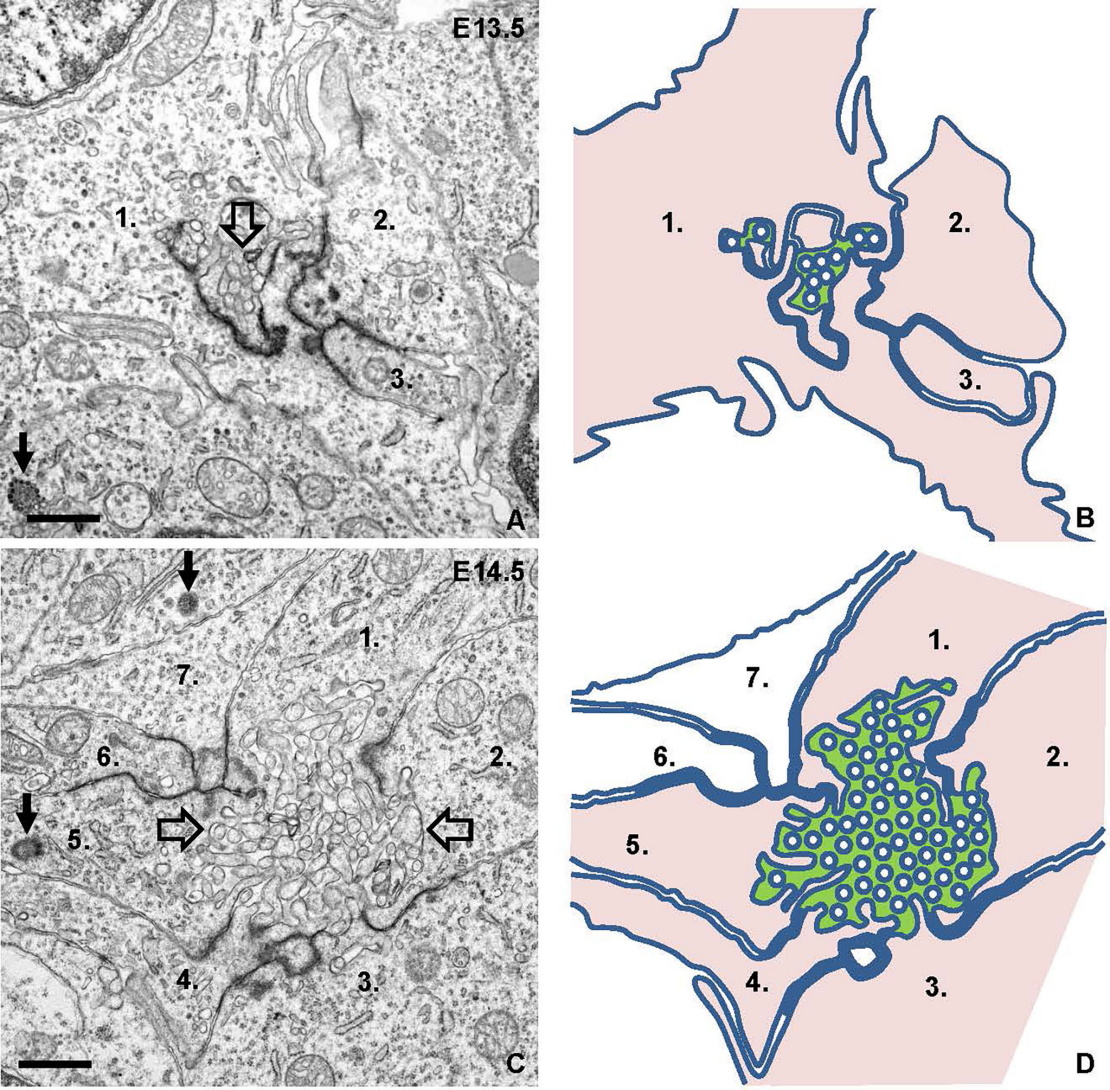
Lumen formation and maturation in thyroid isthmus. Representative transmission electron micrographs from E13.5 **(A)** and E14.5 **(C)** and replicated drawings **(B, D)** of contributing cells. **(A, B)** Microlumen (open arrow) enclosed by elaborate adherens junctions. **(C, D)** Multicellular lumen (open arrows) furnished with apical microvilli. The lumen is green-colored and cells facing the lumen are pink-colored in depicted images for clarity. Numbers (1-3 in **A, B** and 1-7 in **C, D**) indicate individual cell profiles; arrows indicate centrioles. Bars: 1 μm.

**Figure 8:**
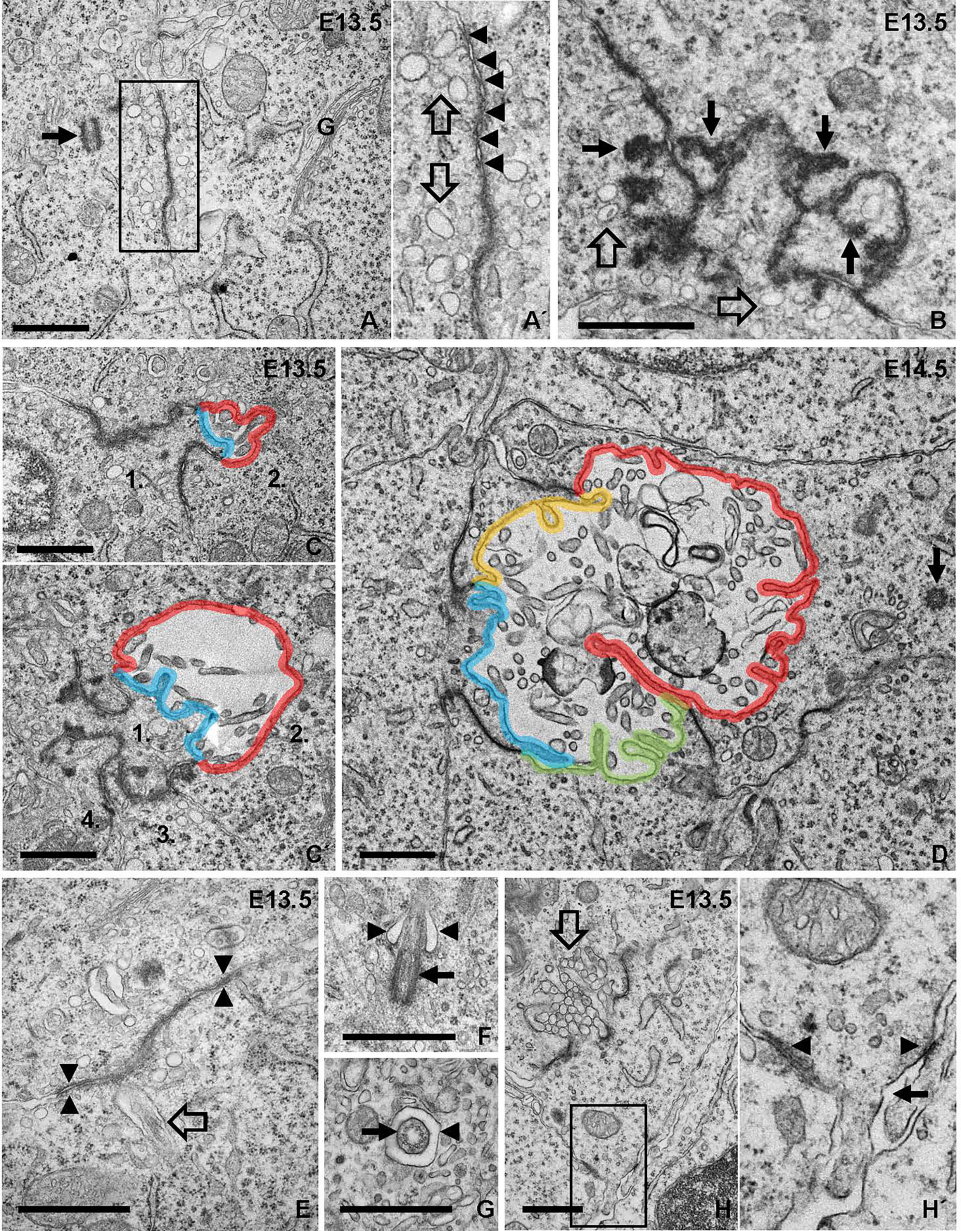
Ultrastructural analysis of apical polarization and lumen initiation. **(A, A´)** Adherence-like junction between progenitor cells. Arrow indicates adjacent centrosome. Inset **(A´)** shows detail of junction with numerous submembranous vesicles (open arrows; also shown in **B**). Arrowheads indicate apposed membrane repeats of junction. **(B)** Irregular junction with club-shaped extensions of electron-dense material projecting cytoplasmically (arrows). **(C, C´)** Bicellular lumen bordered by irregular junctions. Note asymmetric contribution to lumen of cells numbered 1 and 2 with the apical membranes colored blue and red, respectively. **(D)** Lumen maturation involving cell recruitment; each apical membrane colored differently. Arrow indicates centriole in apical cytoplasm. **(E–G)** Ciliogenesis accompanying lumen formation. In **(E)**, rudiment of primary cilium (open arrow) close to adherens-like junction (arrowheads). In **(F)**, ciliary pocket (arrowheads) and basal body (arrow). In **(G)**, cross-sectioned cilium (arrow) in its pocket (arrowhead). **(H, H´)** Focal adhesions of a polarized cell involved in lumen initiation (open arrow). **(H´)** shows high power of inset indicating basolateral cell-cell contacts (arrowheads) and absence of basement membrane (arrow). G, golgi complex. Bars: 1 μm.

Electron microscopy confirmed folliculogenesis taking place already at E13.5 (**Figures 7A, B**). The cells formed tiny, irregular lumens with their apical membranes furnished with microvilli that increased in numbers as more cells participated in follicle development (**Figures 7C, D**). In view of the importance of cell confinement for lumen formation during epithelial morphogenesis *in vitro* (15), we attempted to ultrastructurally identify and characterize cellular changes predicting lumen initiation in the midline thyroid primordium. Based on a comprehensive assessment of numerous conspicuous lumen profiles, this analysis collectively showed that polarizing cells first adhered apically by a junctional stretch of varying complexity (**Figures 8A, B**), which occasionally enclosed a bicellular lumen (**Figures 8C, C´**). Notably, translucent vesicles accumulated in the subjunctional cytoplasm before a discernable microlumen was evident (**Figures 8A´, B**). Moreover, such vesicles did not appear apically in follicular cells forming larger, multicellular lumens (**Figure 8D**), altogether suggesting that a transient pool of vesicles coalesced with the apical membrane for lumen biogenesis. Apical polarization was further documented by the presence of centrioles (**Figures 8A, D**) and signs of ciliogenesis (**Figures 8E–G**) in the apical cytoplasm. Cells triggered to polarize were also connected by focal cell contacts along the basolateral surfaces (**Figures 8H, H´**), which in most instances were devoid of an ultrastructurally discernible basal lamina. These observations thus indicated that most polarizing cells did not yet establish physical contact with a LAM+ basement membrane, which at this developmental stage was restricted to the perimeter of the thyroid primordium (**Figures 3C-C´**).

### Mucin 1 Associates With Centrosomes During Apical Polarization and Lumen Initiation in Embryonic Thyroid Cells

**Figure 9:**
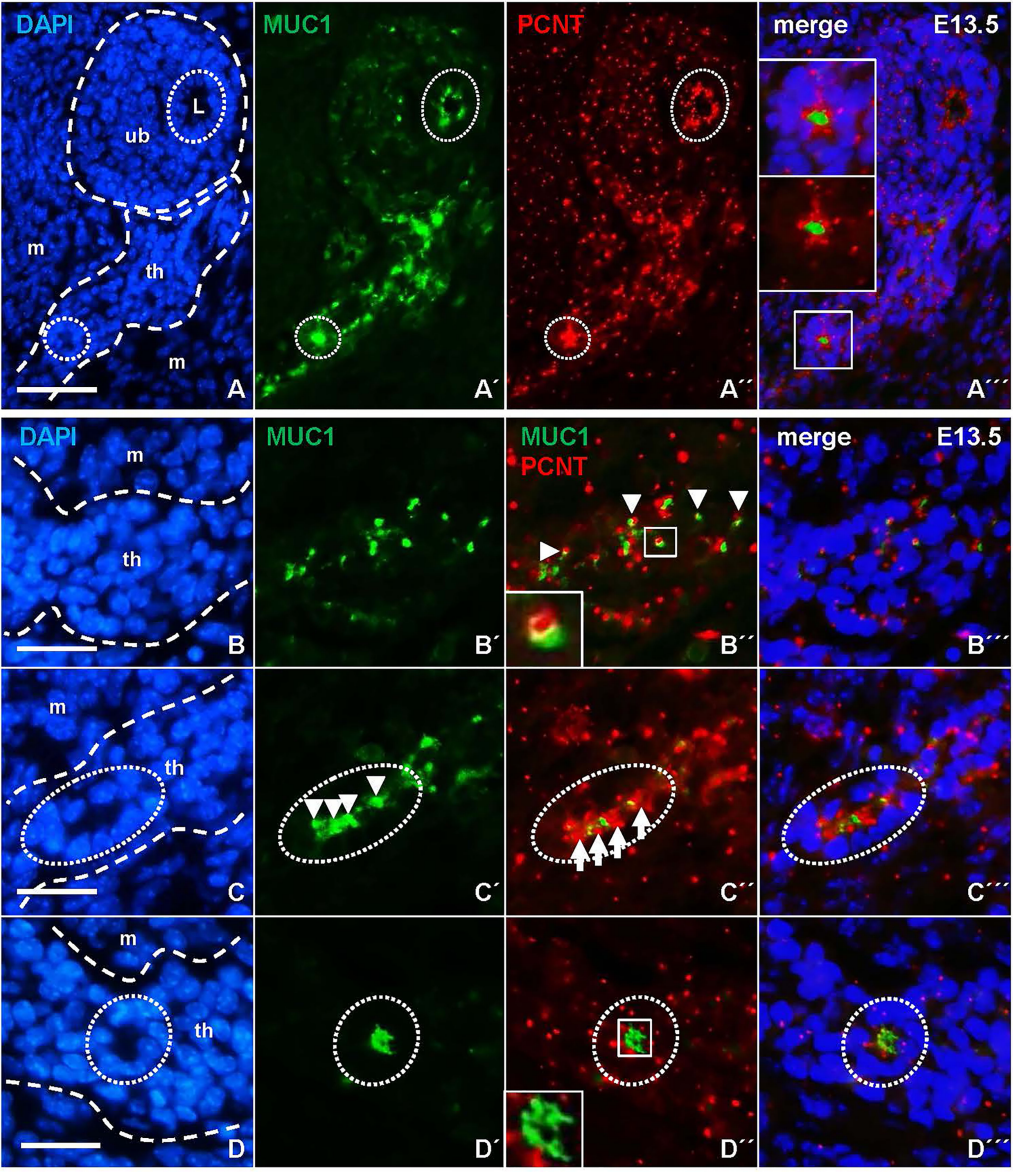
Centrosome-associated assembly of mucin 1 (MUC1) and coalescence of the pre-luminal compartment. Representative data based on detailed evaluation of serially sectioned thyroid tissue from four E13.5 embryos. Images obtained from both single and merged channels (viz. **A´–A´´´**, etc) are shown for clarity. Outer borders of thyroid primordium and ultimobranchial body are outlined in corresponding DAPI images (left panels). **(A)** Associated MUC1 and pericentrin (PCNT) localization in polarized cells. MUC1+ cells facing the lumen (L) of the innermost ultimobranchial epithelium and a presumptive thyroid follicle are encircled for comparison. Insets in **(A´´´)** show high power of boxed area. **(B–D)** Coordinated redistribution of MUC1 and PCNT during apical polarization. **(B´-B´´´)** Colocalization. Arrowheads and inset in **(B´´)** indicate dual-labelled (MUC1+/PCNT+) structures. **(C–C´´´)** Aggregation. Arrows and arrowheads indicate repeats of inwardly oriented centrosomes and MUC1+ structures in a parenchymal cord segment (encircled). **(D–D´´´)** Polarization. Inset in **(D´´)** show coalescence of MUC1+ structures suggestive of lumen initiation. Note apical orientation of centrosomes in lumen-forming cells (encircled). th, thyroid primordium; ub, ultimobranchial body; m, mesenchyme. Bars: 50 µm **(A)** and 25 µm **(B–D)**.

Since MUC1 is expressed during epithelial differentiation of several glandular tissues in both mouse and human embryos (16, 17), we investigated whether the redistribution of MUC1 associated with lumen initiation in the thyroid primordium might correlate to apical polarization of progenitor cells. To this purpose, tissue sections were co-stained for MUC1 and pericentrin (PCNT), a centrosome-associated protein that attains an apical localization in polarized cells (18). This showed that both MUC1 and PCNT were located apically in isthmus cells that faced a presumptive microlumen similar to the features of luminal cells present in the ultimobranchial body, (**Figures 9A-A´´´**), whereas centrosomes in cells that did not participate in follicle formation appeared randomly oriented. However, co-immunostaining revealed that in unpolarized cells MUC1 occasionally associated with PCNT forming a dual complex in which a single MUC1+ particle and the centrosome were closely located but yet distinguishable (**Figures 9B-B´´´** and inset of **B´´**). This differed from polarizing cells in which MUC1+ structures and centrosomes located in the apical cytoplasm were mostly dissociated (**Figures 9C-C´´´**). Centrosomes in cells forming a coalesced MUC1+ compartment designating a definitive site of lumen formation had a free, supranuclear position (**Figures 9D-D´´´**). Collectively, these observations indicate that apical polarization of cells present in the prospective thyroid isthmus involves coordinated association and dissociation of MUC1 and PCNT, which thus might be of importance to establish the pre-follicular phenotype and initiate luminogenesis in the embryonic thyroid.

## Discussion

**Figure 10:**
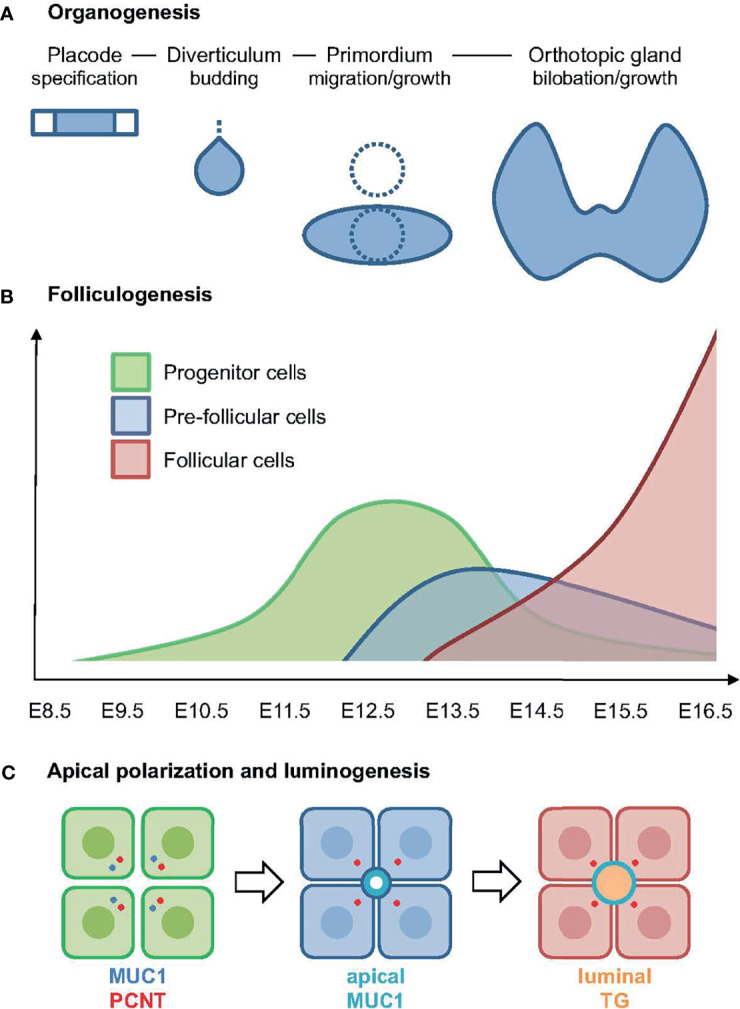
Summary of key findings in the present study. **(A)** Developmental stages of thyroid organogenesis distinguished by morphology and principal morphogenetic features [based on established concepts, for details see (5)]. **(B)** Expansion of progenitors (in green) and onset of folliculogenesis correlated to stages of thyroid development as outlined in **(A)**. The pre-follicular cell population (in blue) expresses mucin 1 (MUC1) and likely represents an intermediate stage of epithelial differentiation towards the complete follicular cell phenotype (in red); x-axis indicates embryonic days after conception. **(C)** Sequence of events in embryonic thyroid cell differentiation: (left panel) progenitor cells start to polarize as revealed by a coordinated redistribution of centrosome-associated pericentrin (PCNT) and MUC1 to the apical cytoplasm; (middle panel) pre-follicular cells jointly establish a MUC1+ apical membrane domain designating lumen initiation; (right panel) follicular cells maintaining MUC1 at the apical surface secrete thyroglobulin (TG) into the lumen.

This study shows that the onset of embryonic thyroid differentiation in mice occurs earlier than previously recognized, and that the process is asynchronous spanning several developmental stages of thyroid organogenesis. The first follicles appeared coinciding with bilateral growth of the midline thyroid primordium, a process by which the isthmus portion is established prior to formation of the left and right lobes (**Figures 10A, B**). Notably, in this intermediate stage thyroid progenitors possess the highest proliferation rate; more than 60% are Ki67+ at E12.5 whereas a minority of cells proliferate in late thyroid development (6, 7). Moreover, primordial growth differs from branching growth of the lobes as proliferating cells in the prospective isthmus are more evenly distributed. Altogether, this suggests that differentiation of embryonic thyroid cells does not necessarily require cessation of growth.

It is well-known from cell culture studies that the expression of thyroid-specific genes and formation of follicles – although being functionally linked in the hormone-producing gland – are independent processes that possess distinct regulatory mechanisms. For example, FRTL5 rat thyroid cells that express all key transcription factors and effector mechanisms required for thyroid hormonogenesis fail to from follicles (19), whereas the sister FRT cell line features the full epithelial repertoire and generate follicle-like cysts, but remains poorly differentiated unable to recover thyroid gene expression even if transfected with a functional TSH receptor (14). Epidermal growth factor (EGF)-stimulated growth of primary thyroid epithelial cells effectively suppresses thyroid function but does not prevent neoformation of follicles in a 3D culture setting (20, 21). The presence of follicles lacking colloid in mice with *Tg* mutations, which confer a misfolded translation product that accumulate in the endoplasmic reticulum (22–24) further argues that folliculogenesis does not require TG, the major secretory product and constituent of luminal colloid. On the other hand, TG-containing exocytic vesicles are the only known source in the mature thyroid gland of membrane additions to the apical plasma membrane that balances endocytosis during stead state turnover of the colloid content (25). Notably, exocytic vesicles transferring TG to the lumen in adult thyroid cells are rapidly mobilized by TSH stimulation and a prerequisite for iodination, which solely takes place extracellularly in the lumen, in normal conditions (26, 27). This relationship thus differs much from the embryonic thyroid in which TG expression and the formation of follicles both are TSH-independent processes (28).

In the present study, we found that MUC1 identifies the earliest stages of *de novo* folliculogenesis featuring lumen initiation, which likely occurred by coalescence of MUC1+ intracellular vesicles accumulating in the apical cytoplasm. Importantly, TG did not co-localize with MUC1 in these pre-follicular cells, indicating that the secretory pathway differed from lumen biogenesis in mouse thyroid progenitors triggered to differentiate (**Figures 10B, C**). In support of this conclusion, as observed by electron microscopy, both the subapical vesicles and microlumens had a translucent interior contrasting to the characteristic electron-dense texture of TG+ exocytic vesicles and luminal colloid in adult thyroid cells (25). As such “empty” apical vesicles were not present in enlarged follicles is consistent with their transient nature being replaced by another population of secretory vesicles by which TG gradually accumulates in the lumen. Folliculogenesis and functional differentiation *in vivo* are thus spatially separated at the cellular level and, although the triggering factor to accomplish embryonic cell maturation might be identical, most likely represent differentially regulated processes (**Figure 10C**). Agreeing with this scenario, *cog/cog* mice do develop follicles but due to the lack of TG secretion the lumens are small and deficient of colloid (22).

MUC1 in humans is mainly of interest concerning the oncogenic splice variant MUC1-C, which is overexpressed in many epithelial cancers including papillary thyroid carcinomas (11, 12). Recent findings indicate that MUC1-C promotes loss of apicobasal polarity and acquisition of an invasive tumor phenotype (29). Moreover, MUC1-C drives tumor progression by influencing lineage plasticity, cell fate and stemness (11, 30, 31). A role of MUC1 in normal organ development is previously suggested by a timely onset of MUC1 expression associated with epithelial polarization and lumen biogenesis during branching morphogenesis (16). The mechanism remains unknown, although it can be speculated that MUC1 might exert an opposite regulatory function to MUC1-C promoting epithelial organogenesis. Our present findings of a MUC1+ vesicular compartment appearing before a MUC1+ microlumen was discernible indicate that MUC1 is not only a biomarker of *de novo* follicle formation but in fact distinguishes thyroid progenitors that start to polarize (**Figure 10C**). Interestingly, MUC1 was similarly distributed in luminal cells of ultimobranchial bodies characterized by lumen involution, suggesting that MUC1 might play an active role in remodeling of tissue architecture during morphogenesis. Dynamic membrane trafficking involving apical endosomes is previously shown for MUC1 in cultured cells (32).

The associated redistribution of MUC1 and PCNT further suggests that the MUC1+ vesicular compartment is dynamically altered along with centrosome displacement during apical polarization of thyroid progenitors (**Figure 10C**). Ultrastructurally, this coincided with the appearance of centrioles and signs of ciliogenesis at sites of lumen initiation, which were easily identified by the abundance of submembranous material reorganizing into typical adherens junctions. This essentially recapitulates the formation of an apical membrane initiation site and a pre-apical patch consisting of two closely interdigitating apical membranes that sequentially occurs before a lumen expands by secretion, as previously characterized in 3D-cultured MDCK cells (15). Notably, the Rho GTPase Ccd42 is required for both centrosome positioning underneath the plasma membrane and recruitment of the junctional protein complex (33, 34), indicating these are coordinated processes. There is consensus that extracellular matrix (ECM) primarily involving laminin exerts a primary apical polarization signal and lumen positioning in epithelial morphogenesis (35). However, of particular interest, a high cell confinement independently promotes centrosome redistribution towards the site of lumen biogenesis, whereas in conditions of low confinement the spreading of cells impairs lumen formation (15). This raises the intriguing possibility that when and where a lumen develops *de novo* during epithelial morphogenesis is spatially controlled by cell density and a constrained ability of progenitor cells to spread and collectively migrate.

In the thyroid primordium at E13.5, most proliferating cells had a random centrosome orientation and were thus lacking an appreciable apical polarity; only a minority of cells jointly participated in follicle formation at this developmental stage. However, there were no obvious differences in the tight packing of cells between follicular and non-follicular portions of the tissue. It was also obvious that not all cells that polarized towards a common MUC1+ pre-luminal compartment had direct contact with a LAM+ basement membrane, which encircled the entire primordium but did not subdivide the tissue interior. It is previously shown that laminin indeed promotes folliculogenesis associated with vascularization of the lobes in later stages of thyroid development (36, 37). Our present findings in the prospective thyroid isthmus suggests that it is not necessary for all cells that polarize and participate in follicle formation to first establish contact with a basement membrane. Lumen initiation, which requires apical polarization of at least two interacting cells, might thus be triggered by a combination of a locally high cell density and laminin deposits, and that further enlargement of the follicle depend on cell division or recruitment of neighboring cells, or both. It is noteworthy that MDCK epithelial cells alternatively orient towards a central lumen or show a collective front-rear polarization typical for motile cells depending on ECM-derived signals that switch mode of intracellular Rho GTPase signaling (38). Translated to the developing thyroid, similar phenotypic changes might take place as the thyroid bud initially disconnects from the pharyngeal endoderm and transitions into a migrating solid structure, after which cells gradually regain apicobasal polarity and folliculogenic potential appearing first within the prospective isthmus portion.

A key question remains to be answered which cues regulate spatiotemporally and finely tunes the onset of functional differentiation during thyroid morphogenesis. In mouse lung development, SOX9 is required to prevent precautious differentiation and thereby promote branching morphogenesis (1, 2). We recently found that, although SOX9 is more strongly expressed in proliferating cells in thyroid lobe development, *Sox9* deficiency neither influenced growth nor differentiation in the embryonic thyroid (7), suggesting that other modifying factors may be more important or redundant for SOX9. Notably, NKX2-1 and PAX8 are co-expressed from the earliest stages of endoderm cells committed to a thyroid fate until the cells become terminally differentiated and onwards to adulthood (13), and a transcriptional network involving both factors regulate thyroid development (39). However, due to developmental arrest already at the bud stage it is not possible to elucidate in knockout mice whether NKX2-1–PAX8 interactions are also required for the later developmental stages. Currently, we merely know that phosphorylation of NKX2-1 coincides with differentiation of the majority of cells during lobe development (40), which suggests that some kinase and/or phosphatase activities are necessary to finalize the developmental program. Whether an altered relative expression of NKX2-1 and PAX8, as suggested from transcript levels monitored between E12.5 and E17.5 in the present study, might be involved in switching embryonic thyroid cells from an immature to a differentiated state remains to be elucidated. It is nevertheless likely that the principal findings of MUC1 expression and its redistribution during apical polarization and luminogenesis are not restricted to isthmus cells but may also account for the bulk of progenitor cells that give rise to all follicles of the lobes. To investigate e.g. by single cell transcriptomics the possibility that induction of cell polarity may influence cell fate decisions and ultimately differentiation, as originally reported for multipotent pancreatic progenitors undergoing tubulogenesis (41), we propose that MUC1 would serve as an ideal biomarker to distinguish the emerging pre-follicular cell population from genuine thyroid progenitor cells, preferentially harvested at a developmental stage prior to full scale folliculogenesis is evident, for expression profiling of transitional phenotypes. Deciphering the transcriptional machinery and regulatory mechanisms that govern apical polarization and functional differentiation *in vivo* may provide new insights for the improved generation of fully functional follicles out of induced pluripotent stem cells.

## Data Availability Statement

The original contributions presented in the study are included in the article/supplementary material. Further inquiries can be directed to the corresponding author.

## Ethics Statement

The animal study was reviewed and approved by Göteborgs djurförsöksetiska nämnd, Kammarrätten i Göteborg, Box 1531, 401 50 Göteborg.

## Author Contributions

EJ, DA, HF, and MN contributed to conception and design of the study. EJ and SL performed animal experiments, microscopy and imaging. CM and TC performed qPCR and DA evaluated qPCR data including statistical analysis. MN wrote the first draft of the manuscript. EJ and DA wrote sections of the manuscript. HF contributed to manuscript revision. All authors contributed to the article and approved the submitted version.

## Funding

The work was supported by the Swedish Research Council (2016-02360 to MN), the Swedish Cancer Society (17-0657 and 20-1279 to MN), the Gothenburg Medical Society (to EJ), Assar Gabrielsson Fund for Cancer Research (to EJ), and Sahlgrenska University Hospital according to the ALF agreement (to HF).

## Conflict of Interest

The authors declare that the research was conducted in the absence of any commercial or financial relationships that could be construed as a potential conflict of interest.

## Publisher’s Note

All claims expressed in this article are solely those of the authors and do not necessarily represent those of their affiliated organizations, or those of the publisher, the editors and the reviewers. Any product that may be evaluated in this article, or claim that may be made by its manufacturer, is not guaranteed or endorsed by the publisher.
